# Examination of the Prefrontal Cortex Hemodynamic Responses to the Fist-Edge-Palm Task in Naïve Subjects Using Functional Near-Infrared Spectroscopy

**DOI:** 10.3389/fnhum.2021.617626

**Published:** 2021-02-05

**Authors:** Satoshi Kobayashi, Yudai Iwama, Hiroshi Nishimaru, Jumpei Matsumoto, Tsuyoshi Setogawa, Taketoshi Ono, Hisao Nishijo

**Affiliations:** ^1^System Emotional Science, Faculty of Medicine, University of Toyama, Toyama, Japan; ^2^Japan Suicide Countermeasures Promotion Center, Tokyo, Japan; ^3^Research Center for Idling Brain Science, University of Toyama, Toyama, Japan

**Keywords:** prefrontal cortex, FEP task, WCST, schizophrenia, fNIRS

## Abstract

The Fist-Edge-Palm (FEP) task, a manual hand task, has been used to detect frontal dysfunctions in clinical situations: its performance failures are observed in various prefrontal cortex (PFC)-related disorders, including schizophrenia. However, previous imaging studies reported that the performance of the FEP task activated motor-related areas, but not the PFC. Here, we aimed to investigate the relationships between the performance of the FEP task and PFC functions. Hemodynamic activity in the PFC, including the dorsolateral PFC (area 46) and frontal pole (area 10), was recorded. Healthy young subjects performed the FEP task as well as a palm tapping (PT) task (control task) three times. The subjects also completed a Wisconsin Card Sorting Test (WCST) and Schizotypal Personality Scale (STA) questionnaire. We found that hemodynamic activity (Oxy-Hb) in the PFC increased in the first trial of the FEP task but decreased considerably in the second and third trials compared to the PT task. The number of performance errors in the FEP task also decreased in the second and third trials. Error reduction (i.e., learning) in the FEP task between the first and second trials was negatively correlated with schizotypal trait and the number of perseveration errors in the WCST. Furthermore, changes in the PFC hemodynamic activity between the first and second trials were positively correlated with error reduction in the FEP task between the first and second trials, and negatively correlated with the number of perseveration errors in the WCST. These results suggest that learning in the FEP task requires PFC activation, which is negatively associated with perseveration errors in the WCST. The results further suggest that the FEP task, in conjunction with near-infrared spectroscopy, may be useful as a diagnostic method for various disorders with PFC dysfunction.

## Introduction

The Fist-Edge-Palm (FEP) task was introduced by Luria (1966) as a sequential movement task to detect frontal dysfunctions. In the FEP task, subjects are asked to successively place their hands on a table in each of the following hand postures: a vertically placed fist (Fist), a vertically placed palm (Edge), and a horizontally placed palm (Palm) (Rao et al., 2008). Luria believed performance impairment of the task to be closely related to contralateral frontal lobe damage (Motomura et al., 1989; Umetsu et al., 2002). Performance impairment of the FEP task was observed not only in deficits in the cortical areas involved in motor control but also in cases of deficits in the prefrontal cortex (PFC) and various neuropsychological disorders with executive dysfunctions such as Parkinson’s disease and schizophrenia (Fama and Sullivan, 2002; Chan and Gottesman, 2008). It has been reported that in cases with frontal lesions, the patients could form each movement pattern of the FEP task independently but could not perform three serial movements (Hayakawa et al., 1999). Based on these findings, the modified FEP and similar serial movement tasks have been widely adopted in clinical practice as standardized tests to assess PFC functions and neurological signs (Chen et al., 1995; Dubois et al., 2000; Chan et al., 2008).

Clinicopathological studies have reported that patients with lesions in the frontal lobe showed performance disturbance of the FEP task (see above). Furthermore, performance deficits in the FEP task in schizophrenia have been ascribed to functional deficits in the PFC (Dazzan et al., 2006; John, 2009; Zhao et al., 2014). These findings suggest that the PFC is required to perform the FEP task. However, two functional magnetic resonance imaging (fMRI) studies reported that the premotor cortex, somatomotor cortex, and supplementary motor area, but not the PFC, were activated by the FEP task in healthy subjects (Umetsu et al., 2002; Chan et al., 2006). In these imaging studies, the subjects were allowed to practice the task before scanning the brain, whereas naïve patients are required to perform the task without prior practice in clinical situations. Thus, the PFC activity may be decreased during fMRI in subjects that have practiced the FEP task before the fMRI (Chan et al., 2006; Rao et al., 2008). Furthermore, previous imaging studies using functional near-infrared spectroscopy (fNIRS) reported that PFC hemodynamic activity increased during the learning of hand dexterity tasks (Ishikuro et al., 2014; Ota et al., 2020). Therefore, we hypothesized that the PFC hemodynamic activity would increase when naïve subjects undertake the FEP task. fNIRS can be applied in conditions similar to clinical situations, with the body and head relatively movable compared to an fMRI environment (Okamoto et al., 2004; Ishikuro et al., 2014). In the present study, we applied fNIRS to assess the PFC hemodynamic responses to the FEP task in naïve subjects.

## Materials and Methods

### Subjects

Nineteen healthy subjects who did not have any physical or neurological problems participated in this study (mean age, 23.00 ± 0.30 years; ten females, nine males; all right-handed). The study was conducted in accordance with the Declaration of Helsinki and the United States Code of Federal Regulations for the protection of human subjects. The experimental protocol was approved by the ethical committee at the University of Toyama, and written informed consent was obtained from all subjects before the initiation of the experiments.

The subjects sat down in a chair toward a table and initially completed a self-report Oxford Schizotypal Personality Scale (STA) questionnaire (Japanese version; Ueno et al., 2010; Itaguchi et al., 2018). They then underwent the Wisconsin Card Sorting Test (WCST) on a computer display (Banno et al., 2012; software available^1^). Then, a head cap for the fNIRS recording (Shimadzu Co. Ltd., Japan) was placed on the head of the subjects, and they received instructions on how to perform the manual hand movement tasks via video. Following this, the subjects performed the manual hand movement tasks while their cerebral hemodynamic activity was recorded using fNIRS.

### Wisconsin Card Sorting Test (WCST)

On the computer display, a target card and four stimulus cards with pictures that varied in shape, color, and number were displayed. The subject was asked to select one of the four stimulus cards based on one of the three dimensions that appeared to match the target card. After each response of the subjects, feedback on the correctness of the response was provided to the subjects. After six successive correct responses, the matching rule changed, and the subjects were informed of the rule change. A total of 48 trials were performed for each subject.

### Manual Hand Movement Tasks

**FIGURE 1:**
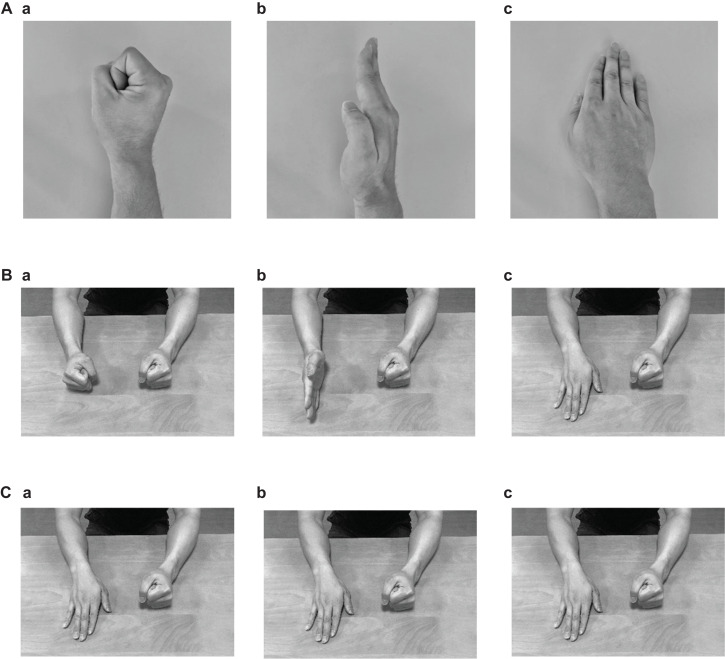
Photographs indicating the postures of the right hand **(A)** and both arms **(B)** in the Fist-Edge-Palm (FEP) task, and postures of both arms in the palm tapping (PT) task **(C)**. **(Aa–c)** Photographs showing vertical “fist” **(a)**, vertical palm (i.e., “edge”) **(b)**, and horizontal “palm” **(c)** on a table in the FEP task. **(Ba–c)** Photographs showing both arms corresponding to **(Aa–c)**. **(Ca–c)** Palm tapping on a table in the PT task.

The subjects conducted the FEP and palm tapping (PT) tasks that were applied by Chan et al. (2006). In the FEP task, the subjects were required to sequentially place their right hand with each specific positioning (fist, edge, or palm) on the table (Figures 1A,B) (Chan et al., 2006). As a control task (PT task), the subjects were asked to tap on the table with their right palm repeatedly (Figure 1C).

Each subject was provided with video instructions about the experiment and tasks, in which a model performed each task (FEP or PT). One session for each task consisted of three trials. The order of the tasks was randomized among the subjects to avoid an order effect. One trial began with a 30 s resting period followed by the execution of each task for 30 s and another resting period for 30 s. The subjects were instructed to perform the FEP and PT tasks of three movements/s at a constant pace under the guidance of metronome sounds (Tempo-Metronome, Frozen Ape Pte. Ltd., Singapore).

### fNIRS Recording

**FIGURE 2:**
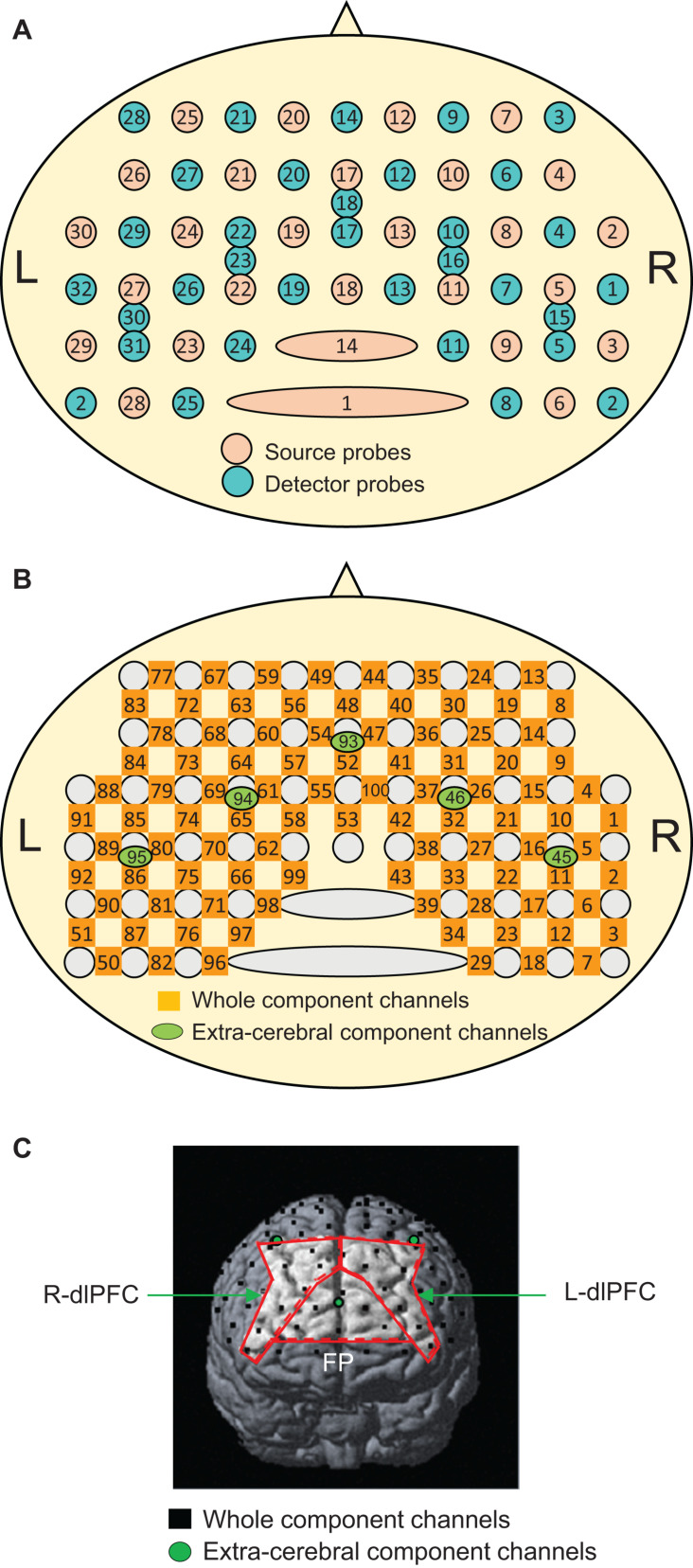
Arrangement of the near-infrared spectroscopy (NIRS) source and detector probes **(A)** and channels **(B)**, and an example of the distribution of NIRS channels in one subject **(C)**. The brain regions surrounded by the red lines indicate the three ROIs: the frontal pole (FP) area, and right (R-dlPFC), and left (L-dlPFC) dlPFC. In panel **(C)**, only three short channels (green circles) are shown. The remaining two short channels are located posteriorly behind the outer edge of the cerebral cortex and are invisible.

The recording methods used were essentially similar to our previous fNIRS studies (Ishikuro et al., 2014; Nakamichi et al., 2018; Hibi et al., 2020; Ota et al., 2020). Briefly, the subjects were seated in a chair facing toward a table, and a head cap to fix fNIRS probes (Shimadzu Co. Ltd., Japan) was placed on the head of the subject. Most anterior probes were set along the FP10-FP2 line of the electroencephalogram (EEG) 10–20 system for electrode positioning (Jurcak et al., 2007). Two fNIRS systems (OMM3000, Shimadzu Inc., Kyoto) were combined to measure the hemodynamic activity in the bilateral frontal lobes. A total of 30 sources and 32 detector probes were arranged on the same head cap as in our previous study (Figure 2A) (Hibi et al., 2020). Three different wavelengths (780 nm, 805 nm, and 830 nm) were emitted from the source probes and detected by the detector probes to measure hemodynamic responses, where changes in Hb concentration (Oxy-Hb, Deoxy-Hb, and Total-Hb [Oxy-Hb + Deoxy-Hb]) were computed according to the modified Beer–Lambert law (Seiyama et al., 1988; Wray et al., 1988).

Hemodynamic signals include different source signals depending on the probe distance between the source and detector probes (Fukui et al., 2003; Niederer et al., 2008; Ishikuro et al., 2014). Hemodynamic signals include cerebral (brain) and extracerebral (scalp, skull, and cerebrospinal fluid) components when the probe distance is more than 3 cm, while the signals mainly reflect extracerebral components when the probe distance is less than 1.5 cm. In the present study, a multi-distance probe arrangement was applied. The 27 detector probes were positioned at a distance of 3 cm from the source probes, while another five detector probes were positioned 1.5 cm from the source probes (Figure 2A). The midpoints between the pairs of source and detector probes with 3 cm distance and signals from those probes were registered as “whole component channels” and “whole signals,” respectively. A total of 95 whole component channels and the corresponding whole signals were registered (Figure 2B). The midpoints between the pairs of probes with a 1.5 cm distance and signals from those probes were registered as “extracerebral component channels” and “extracerebral signals,” respectively. A total of five extracerebral channels and corresponding extracerebral signals were analyzed (Figure 2B). After the experiments, the three-dimensional (3D) locations of the probes were measured in each subject using a digitizer (FASTRAK, Polhemus Inc., United States) to estimate the 3D locations of the channels.

### Data Analysis

#### Psycho-Behavioral Data

In the STA, the total score was estimated for each subject. In the WCST, the number of achieved categories, number of total errors, number of Milner type perseveration errors (errors in which subjects matched the target cards according to the correct matching rule before the rule changed; Milner, 1963), and the number of Nelson type perseveration errors (errors in which subjects matched the target cards based on a wrong matching rule in the immediately preceding trial; Nelson, 1976) were counted for each subject.

The performance of each subject in the manual hand movement tasks was videotaped (Everio GZ-MG275, Victor/JVC) and later examined offline. We defined task execution errors when the tempo of movements changed, the sequence of movement actions was incorrect, or the movements stopped (Chen et al., 1995). Thus, the following hand actions were counted as errors: (1) stop of hand action, (2) formation of a wrong hand shape, (3) putting the hand on the table with an incomplete hand placement, (4) incomplete control of the pronation/supination motion of the hand, and (5) hand placement in one position for 0.5 s or more. When an incorrect hand action satisfied more than two of the above criteria, it was counted as one error. When incorrect hand actions were observed after stopping the hand actions or after the re-formation of a correct hand action, these hand actions were counted as independent errors. The number of these errors was used as an index of task performance failure.

The number of errors across three trials in the FEP task was analyzed using the Wilcoxon rank-sum test due to their non-normal distribution. Relationships between parameters in the behavioral tasks were analyzed using a linear regression analysis.

#### fNIRS

The NIRS signals were initially processed to remove systemic components. NIRS signals from the short distance (1.5 cm) probes are supposed to predominately include systemic components of hemodynamic activity in the scalp and skull. A spatial filter algorithm based on the principal component analysis (PCA) of five extracerebral component signals (Zhang et al., 2016, 2017) was used to remove systemic components from the whole component signals. The first two principal components derived from the PCA of the data of these five extracerebral component signals were removed from the whole component signals. The resultant NIRS signals were bandpass filtered at 0.01 Hz to 0.1 Hz to remove physiological noises derived from respiration, cardiac activity, and baseline drifts (Tong et al., 2011; Yasumura et al., 2014). The above data analyses were performed using commercial NIRS analysis software (ADVANCED ROI) (WAWON DIGITECH, Japan).

In the present study, Oxy-Hb data were further analyzed because the signal-to-noise ratio of Oxy-Hb was larger than Deoxy-Hb (Sato et al., 2016). Furthermore, Deoxy-Hb is sensitive to not only venous blood oxygenation but also to other factors such as venous blood volume (Hoshi et al., 2001). The resultant NIRS signals in each trial were corrected for baseline activity for 15 s before the start of the task. Then, Oxy-Hb responses during the tasks were converted to effect sizes. The effect sizes of the hemodynamic responses during the tasks were computed as follows: effect size = ([mean Oxy-Hb levels for 40 s after the task onset] – [mean Oxy-Hb levels during the baseline period for 15 s before the task onset])/(standard deviation of Oxy-Hb levels during the baseline period for 15 s before task onset).

The anatomical location of each channel was determined by converting the 3D locations of the NIRS channels to the MNI coordinates in each subject using virtual registration by NIRS SPM (statistical parametric mapping) software^2^ (version 3.1) within MATLAB (R2007b) (Ye et al., 2009). The identification of the anatomical locations of the NIRS channels based on the Brodmann areas (BA) was performed in each subject using MRIcro software^3^ (Figure 2C). We focused on three brain regions for the regions of interest (ROIs), the frontal pole (area 10), and the bilateral dorsolateral PFC (dlPFC, area 46). In each subject, the mean effect size of the Oxy-Hb responses was estimated in each ROI.

#### Statistical Analyses

Data normality was checked using the Shapiro–Wilk test. Homogeneity of variance was checked using Levene’s test. The effect sizes in the three ROIs during the tasks were analyzed using a repeated-measures three-way analysis of variance (ANOVA) with the ROI, task, and trial of the task as factors with *post hoc* tests (Bonferroni test). The relationship between the mean PFC hemodynamic responses across the three ROIs and psycho-behavioral data were analyzed using a linear regression analysis and Spearman’s rank correlation test. A *p*-value of 0.05 was considered statistically significant. SPSS statistics version 17 (SPSS Inc., Chicago, IL, United States) was used for the statistical analyses.

## Results

### Psycho-Behavioral Data

The mean total score in the STA was 9.15 ± 1.43 (mean ± SEM). In the WCST, the behavioral data were as follows: the number of achieved categories, 5.47 ± 0.17; the number of total errors, 12.21 ± 0.53; the number of Milner type perseveration errors, 0.78 ± 0.25; and the number of Nelson type perseveration errors, 1.10 ± 0.33. There were no significant gender differences in STA scores (Mann–Whitney *U* test, *p* = 0.512), number of achieved categories (Mann–Whitney *U* test, *p* = 0.639), number of total errors (*t*-test, *p* = 0.447), number of Milner type perseveration errors (Mann–Whitney *U* test, *p* = 0.500), nor number of Nelson type perseveration errors (Mann–Whitney *U* test, *p* = 0.276).

**FIGURE 3:**
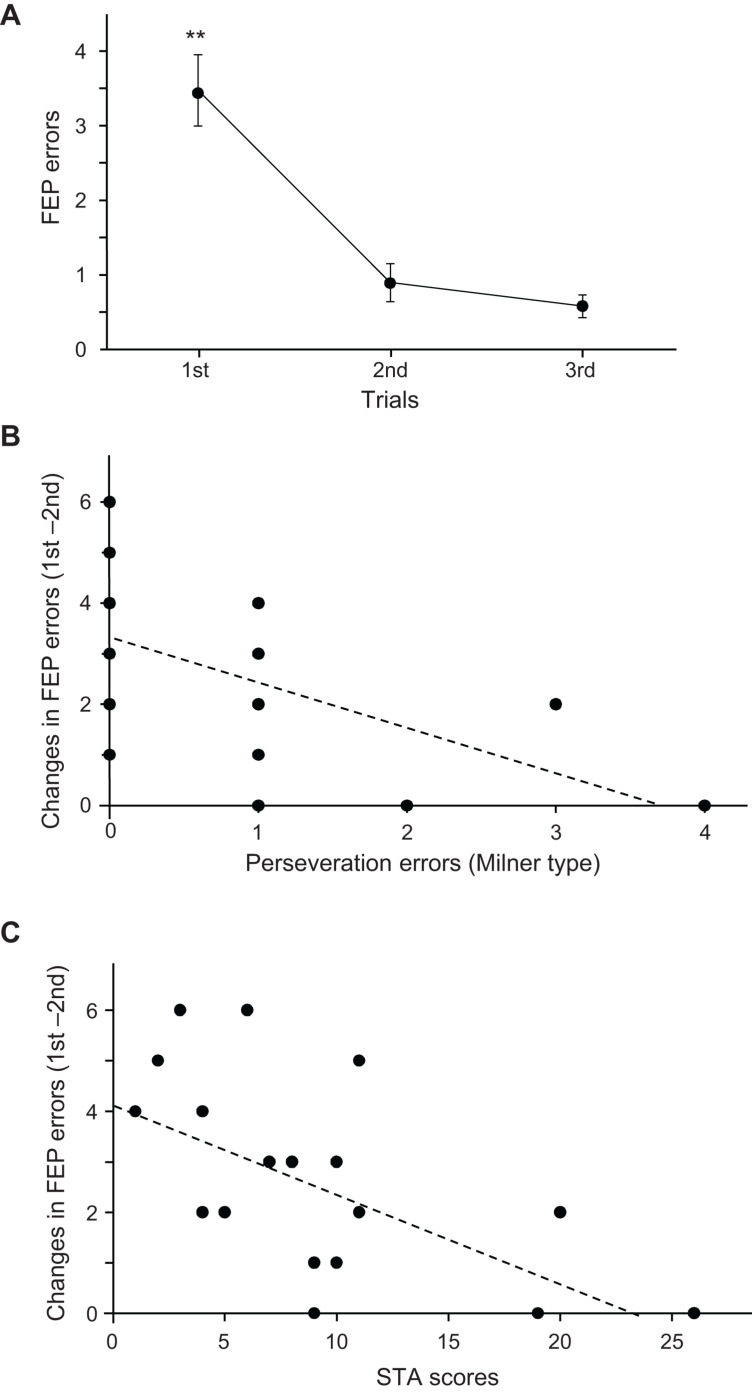
Relationships among the psycho-behavioral data in the Fist-Edge-Palm (FEP) task, Wisconsin Card Sorting Test (WCST), and Schizotypal Personality Scale (STA) total scores. **(A)** Number of errors across the three trials in the FEP task. **Significant difference from the second and third trials (*p* < 0.01). **(B)** Relationship between the changes in errors between the first and second trials in the FEP task and Milner type perseveration errors in the WCST. Each dot indicates each subject; the total number of dots is 19, but some dots overlap. **(C)** Relationship between changes in the errors between the first and second trials in the FEP task and total scores in the STA.

Figure 3A shows the mean number of errors across the three trials in the FEP task. Since the data in the second and third trials did not show normal distribution, the data were compared using the Wilcoxon rank-sum test. The mean number of errors in the FEP task were significantly larger in the first trial than in the second (*p* = 0.001) and third trials (*p* < 0.0001).

Figure 3B shows the relationship between the number of Milner type perseveration errors in the WCST and changes in the number of errors between the first and second trials (number of errors in the first trial minus that in the second trial) in the FEP task. A linear regression analysis indicated that the error reduction in the FEP task was significantly and negatively correlated with the number of perseveration errors in the WCST (*r* = −0.553; *F*[1,18] = 7.493, *p* = 0.014). Spearman’s rank correlation test also revealed a significant negative correlation between the error reduction in the FEP task and the number of perseveration errors in the WCST (rs = −0.630, *p* = 0.004). These results indicate that a larger reduction of errors in the FEP task is associated with fewer perseveration errors in the WCST. Figure 3C shows the relationships between the STA scores and changes in the number of errors between the first and second trials in the FEP task. A linear regression analysis indicated that the error reduction in the FEP task was significantly and negatively correlated with the STA scores (*r* = −0.582; *F*[1,18] = 8.714, *p* = 0.009). Spearman’s rank correlation test also revealed a significant negative correlation between the error reduction in the FEP task and the STA scores (rs = −0.582, *p* = 0.009). These results indicate that a larger reduction of errors in the FEP task is associated with lower STA scores. However, there were no significant relationship between the STA scores and the number of perseveration errors in the WCST (*r* = 0.035; *F*[1,18] = 0.021, *p* = 0.886). Spearman’s rank correlation test also revealed that there was no significant correlation between the STA scores and the number of perseveration errors in the WCST (rs = 0.130, *p* = 0.596).

### Hemodynamic Activity in the Manual Hand Movement Task

**FIGURE 4:**
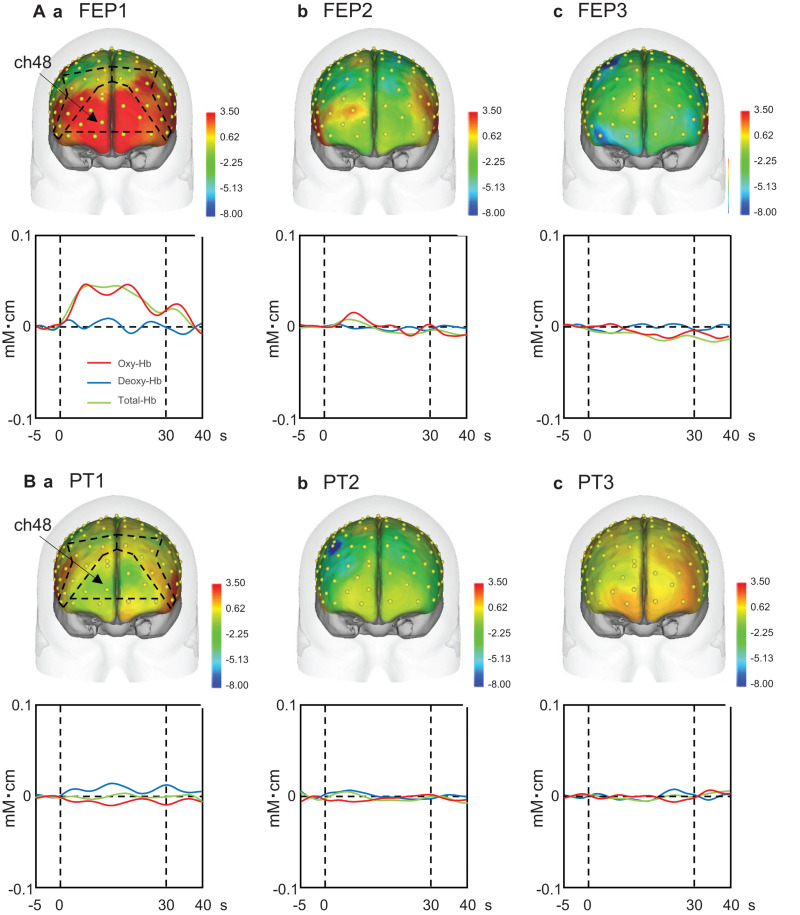
Examples of the hemodynamic responses in the prefrontal cortex (PFC) across the three trials in the Fist-Edge-Palm (FEP; **A**) and palm tapping (PT; **B**) tasks. Topographical maps indicate the effect sizes of the hemodynamic (Oxy-Hb) responses during the tasks. FEP1-3, first **(Aa)**, second **(Ab)**, and third **(Ac)** trials in the FEP task; PT1-3, first **(Ba)**, second **(Bb)**, and third **(Bc)** trials in the PT task. Yellow dots on the head indicate the whole component channels. ch, NIRS channel number. Insets indicate temporal changes in the hemodynamic activity in ch 48 during the FEP and PT tasks. Red, blue, and green lines in insets indicate Oxy-Hb, Deoxy-Hb, and Total-Hb, respectively.

Upper panels in Figure 4A shows examples of the topographical maps of the hemodynamic responses (effect sizes) in the PFC across the three trials of the FEP task. These maps indicate that hemodynamic responses increased in the frontal pole (FP) andright and left dlPFC in the first trial, but not in the second and third trials. Lower panels Figure 4A shows representative temporal patterns of the Oxy-Hb signals recorded from a center of the PFC (ch 48), which showed similar changes. Oxy-Hb signals increased in the PFC only in the first trial, whereas such hemodynamic activity was not evident in the second and third trials of the FEP task. In the PT task (Figure 4B), hemodynamic activity (ch 48) was not evident in the PFC across the three trials.

**FIGURE 5:**
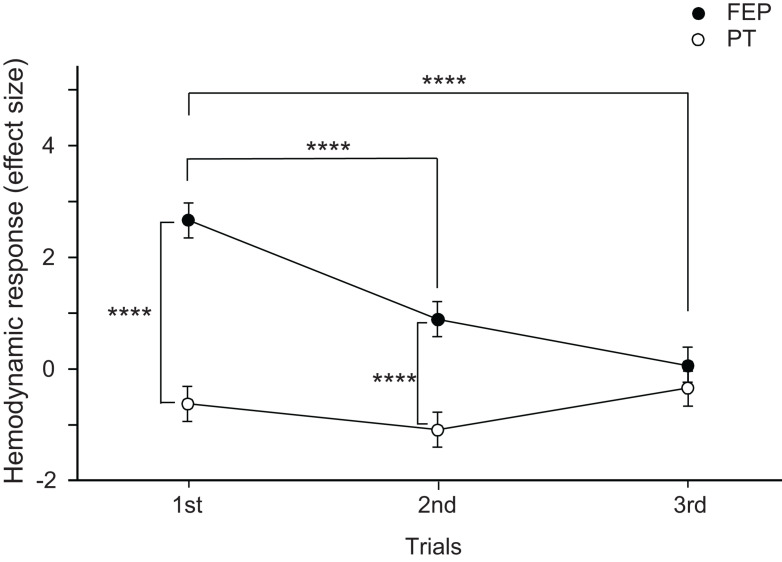
Comparison of the hemodynamic responses (effect sizes of Oxy-Hb responses) in the prefrontal cortex (PFC) between the Fist-Edge-Palm (FEP) and palm tapping (PT) tasks. ****p < 0.0001.

Mean effect sizes in the three ROIs were analyzed using a repeated-measures three-way ANOVA with the ROI (FP vs. right dlPFC vs. left dlPFC), task (FEP vs. PT), and trial as factors. The statistical results indicate significant main effects of task (*F*[1,324] = 48.771, *p* < 0.0001) and trial (*F*[2,324] = 10.612, *p* < 0.0001), and a significant interaction between task and trial (*F*[2,324] = 11.791, *p* < 0.0001), whereas the main effect of the ROI (*F*[2,324] = 0.263, *p* = 0.769) and all remaining interactions between or among the ROIs and other factors were not significant (data not shown). These data indicate that there were no differences among the three ROIs. Therefore, effects of an interaction between task and trial on effect sizes were analyzed (Figure 5). The *post hoc* comparisons indicate that the mean effect sizes were significantly greater in the FEP task than those in the PT task in both the first (Bonferroni test, *p* < 0.0001) and second (Bonferroni test, *p* < 0.0001) trials. Furthermore, the mean effect sizes in the FEP task were significantly greater in the first trial than in the second and third trials (Bonferroni test, *p* < 0.0001).

We also analyzed gender effects on hemodynamic activity. The above results indicate that there were no significant differences among the three ROIs. Therefore, the mean effect sizes across the three ROIs were analyzed by repeated-measures three-way ANOVA with gender, task and trial as factors. The statistical results indicate significant main effects of trial (*F*[2,102] = 3.689, *p* = 0.028) and task (*F*[1,102] = 23.89, *p* < 0.0001), and a significant interaction between task and trial (*F*[2,102] = 4.527, *p* = 0.013), whereas the main effect of gender (*F*[1,102] = 0.391, *p* = 0.533) and all remaining interactions between or among gender and other factors were not significant (data not shown). These results indicate no gender effects on PFC hemodynamic activity in the FEP and PT tasks.

### Relationships Between the Behavioral Data and PFC Hemodynamic Responses

**FIGURE 6:**
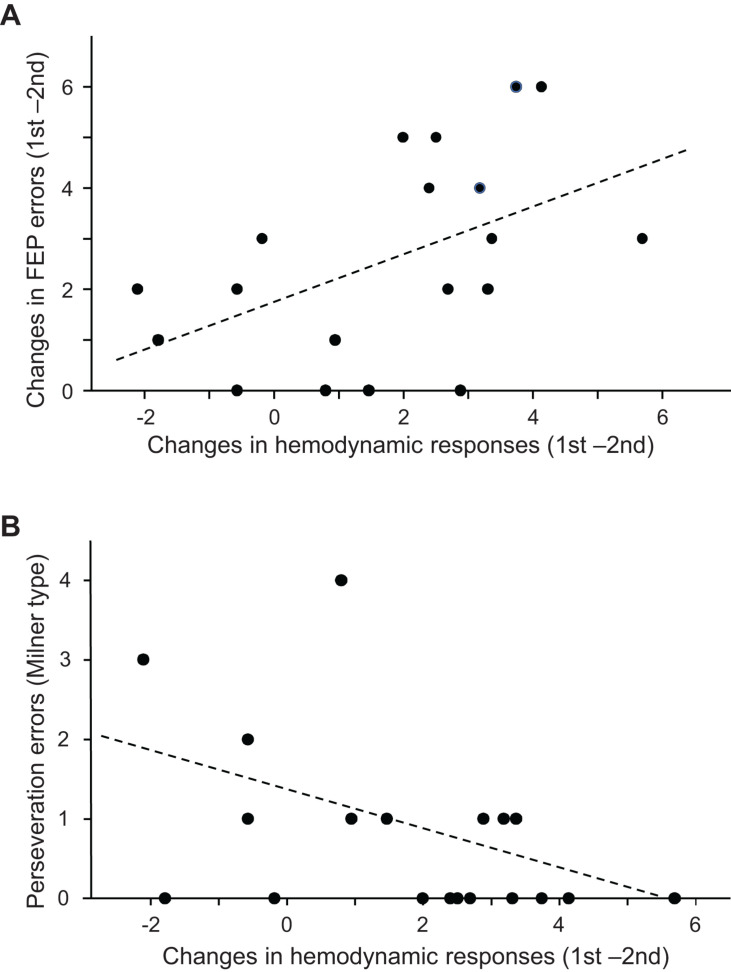
Relationships between the prefrontal cortex (PFC) hemodynamic responses in the Fist-Edge-Palm (FEP) task and psycho-behavioral data. **(A)** Relationship between the changes in the PFC hemodynamic responses (effect sizes) between the first and second trials and error reduction between the first and second trials in the FEP task. **(B)** Relationship between the changes in the PFC hemodynamic responses (effect sizes) between the first and second trials of the FEP task and Milner type perseveration errors in the Wisconsin Card Sorting Test (WCST).

The above analyses indicated that both the performance errors in the FEP task and mean hemodynamic responses in the PFC decreased in the second trial compared to those in the first trial. Figure 6A shows the relationships between error reduction in the FEP task between the first and second trials (i.e., behavioral changes due to learning) and PFC hemodynamic response changes between the first and second trials (i.e., hemodynamic response in the first trial minus that in the second trial) analyzed using a linear regression analysis. The error reduction was significantly and positively correlated with hemodynamic response (effect size) changes (*r* = 0.490; *F*[1,18] = 5.360, *p* = 0.033). Spearman’s rank correlation test also revealed a significant positive correlation between the error reduction and the hemodynamic responses (rs = 0.536, *p* = 0.018). This indicates that a larger error reduction is associated with larger hemodynamic response changes in the PFC.

Figure 6B shows the relationships between the number of Milner type perseveration errors in the WCST and the PFC hemodynamic response (effect size) changes between the first and second trials in the FEP task. A linear regression analysis indicated that the number of Milner type perseveration errors were significantly and negatively correlated with hemodynamic response changes between the first and second trials (*r* = −0.461; *F*[1,18] = 4.598, *p* = 0.047). Spearman’s rank correlation test also revealed that the number of Milner type perseveration errors tended to be negatively correlated with hemodynamic response changes between the first and second trials (rs = −0.446, *p* = 0.055). In the PT task, there was no significant correlation between the number of Milner type perseveration errors in the WCST and the hemodynamic response changes between the first and second trials (linear regression: *r* = −0.156, *F*[1,18] = 0.421, *p* = 0.525; Spearman’s rank correlation test: rs = −0.120 *p* = 0.625). Furthermore, there was no significant correlation between the number of Milner type perseveration errors in the WCST and the differences in hemodynamic responses between the FEP and PT tasks (hemodynamic response changes between the first and second FEP trials - those between the first and second PT trials (linear regression: *r* = −0.315; *F*[1,18] = 1.874, *p* = 0.189; Spearman’s rank correlation test: rs = −0.299 *p* = 0.213). These findings indicate that the subjects with larger hemodynamic response changes between the first and second trials in the FEP task displayed fewer perseveration errors in the WCST. In addition, the number of Nelson type perseveration errors in the WCST was significantly and negatively correlated with PFC hemodynamic response changes between the first and second trials (Supplementary Figure 1A), and the number of total errors in the WCST tended to be negatively correlated with PFC hemodynamic response changes between the first and second trials (Supplementary Figure 1B). On the other hand, the number of achieved categories in the WCST was positively correlated with PFC hemodynamic response changes between the first and second trials (Supplementary Figure 2).

However, there were no significant linear relationships between the STA scores and PFC hemodynamic activity changes between the first and second trials in the FEP task (*r* = −0.115; *F*[1,18] = 0.228, *p* = 0.639). Spearman’s rank correlation test also revealed no significant correlation between the STA scores and PFC hemodynamic activity changes between the first and second trials in the FEP task (rs = −0.326, *p* = 0.173). In the PT task, there was no significant correlation between the STA scores and the PFC hemodynamic response changes between the first and second trials (linear regression: *r* = −0.238; *F*[1,18] = 1.025, *p* = 0.325; Spearman’s rank correlation test: rs = −0.048, *p* = 0.844). Furthermore, there was no significant correlation between the STA scores and the differences in PFC hemodynamic responses between the FEP and PT tasks (hemodynamic response changes between the first and second FEP trials - those between the first and second PT trials) (linear regression: *r* = −0.041; *F*[1,18] = 0.029, *p* = 0.866; Spearman’s rank correlation test: rs = −0.190, *p* = 0.436).

## Discussion

### Comparison With Previous Studies on FEP Task

To examine PFC involvement in the execution of the FEP task, we analyzed brain activation during the performance of the FEP and PT tasks in healthy subjects. Our results indicate that all three ROIs in the PFC were significantly more activated during the FEP than PT tasks. However, this difference was dependent on the number of trials. Hemodynamic activity in the FEP task was significantly decreased in the second and third trials compared to the first trial, and there was no difference in hemodynamic activity between the FEP and PT tasks in the third trial.

Previous fMRI studies have reported that the bilateral sensorimotor cortex, bilateral premotor areas, supplementary motor area, left parietal lobe, and right cerebellum were more activated in the FEP task than in the PT task and that the FEP task required a wider range of brain activation than simple PT or control tasks (Umetsu et al., 2002; Chan et al., 2006, 2015b; Rao et al., 2008). However, PFC activation was not observed in the FEP task in these studies, although functional connectivity between the PFC and sensorimotor cortex was increased during the FEP task, suggesting a regulatory role of the PFC in the FEP task (Rao et al., 2008; Chan et al., 2015b). In this study, using NIRS, which can be applied to clinical practice under similar conditions in naïve subjects, we found that the FP and bilateral dlPFC were activated, especially during early trials in the FEP task. The present results are consistent with those of previous studies in that there were no significant differences in PFC activity in the third trial in this study. The results of the subjects that received training for the FEP task before fMRI scanning in previous fMRI studies (Umetsu et al., 2002; Chan et al., 2006, 2015b; Rao et al., 2008) might be comparable to the results from the third trials of the FEP task in the present study. Furthermore, we imposed a faster rate of the FEP task execution (i.e., 180 bpm) than that imposed in previous studies (Rao et al., 2008; Chan et al., 2015b: 60 or 90 bpm). This may have made the FEP task more challenging, which may have helped to activate the PFC.

### Role of the PFC in Manual Hand Control

The present study indicated that PFC activity was increased, especially in the first trial of the FEP task. Furthermore, reduction of the performance errors in the FEP task was significantly associated with PFC hemodynamic responses (changes in hemodynamic responses between the first and second FEP trials). A previous study suggested that the PFC shapes synaptic activity in the hand area of the primary motor (MI) cortex so that MI activity is optimal for manual hand actions (Ota et al., 2020). These findings suggest that error reduction in the FEP task between the first and second trials (i.e., behavioral changes due to learning) might be attributed to hemodynamic response changes between the first and second FEP trials. Accumulating evidence has reported PFC involvement in manual hand control. Monkey behavioral studies have reported that lesions of the dlPFC altered behaviors in a manual hand dexterity task (spatio-temporal sequence to grasp pellets from differently located wells) and decreased grip force, and the impact of the lesions was greater in larger dlPFC lesions (Kaeser et al., 2013; Badoud et al., 2017). Human fMRI studies have reported that the PFC, including the dlPFC, is activated in precision grip tasks using the right hand (Ehrsson et al., 2001; Vaillancourt et al., 2007; Wasson et al., 2010; Neely et al., 2013). In the monkey behavioral studies (Badoud et al., 2017), the effects of PFC lesion sides were variable, with the effects of the unilateral dlPFC lesions observed in both hands, the ipsilateral hand, or contralateral hand. Consistently, the activated sides in human fMRI studies were variable across studies. These studies reported that the ipsilateral, contralateral, or bilateral PFC was activated during manipulation of the right hand (Ehrsson et al., 2001; Vaillancourt et al., 2007; Wasson et al., 2010; Neely et al., 2013). In the present study, the bilateral dlPFC was activated. These variable effects might be attributed to the dlPFC anatomical connections to the frontal motor areas, including the premotor cortex, supplementary motor area, and cingulate motor area (Morecraft et al., 2012; Pineda-Pardo et al., 2019; Schulz et al., 2019), which control the bilateral hands (Kermadi et al., 1998, 2000).

In the present study, the FP (area 10) was also activated, especially in the first trial of the FEP task. Several previous studies have reported FP involvement in motor control. A human neurophysiological study with deep EEG recordings reported that functional coupling based on gamma-band oscillation between the FP and hand muscle increased during intentional hand muscle contraction (Babiloni et al., 2008). Furthermore, the FP was activated during the learning of new motor task(s) (Jenkins et al., 1994; Floyer-Lea and Matthews, 2004), while patients with lesions in the PFC, including the FP, showed deficits in motor learning (de Guise et al., 1999; Richer et al., 1999). The FP projects not only to the dlPFC but also to the medial PFC, including the anterior cingulate cortex and the striatum (Carmichael and Price, 1996; Petrides, 2005; Petrides and Pandya, 2007), which are all implicated in motor control and learning (Halsband and Lange, 2006). Furthermore, the FP, premotor area, and primary motor cortex are interconnected by multi-synaptic pathways (Petrides, 2005). These findings suggest that the FP controls hand motor behaviors through these motor-related areas.

### Relationships Between the PFC Hemodynamic Activity and Psycho-Behavioral Data

In the present study, fewer perseveration errors in the WCST were associated with larger error reduction between the first and second trials in the FEP task. This suggests that error reduction between the first and second trials in the FEP task reflects executive functions. Furthermore, larger changes in the PFC hemodynamic responses between the first and second trials were associated with larger error reduction between the first and second trials of the FEP task and also associated with fewer perseveration errors in the WCST. These results suggest that error reduction in the FEP task was attributed to PFC executive functions. Consistently, the dlPFC is reported to be an essential brain region for executive functions, especially behavioral and cognitive flexibility (Tei et al., 2017). The FP has also been reported to be active during task switching in which task rules or paradigms changed (Braver et al., 2003; Asari et al., 2005) and also active across different tasks (Sakai and Passingham, 2003). Furthermore, the FP appears to be involved in the inhibition of interference from a previous task rule (Konishi et al., 2005). These findings support Luria’s assumption that the execution of the FEP task requires PFC functions in naïve subjects.

Clinical studies have reported that patients with schizophrenia show higher neurological soft signs (NSS), including performance deficits in the FEP task (Chan and Gottesman, 2008; Chan et al., 2010, 2015a). Patients with schizophrenia show PFC morphological and functional abnormalities, which are associated with deficits in executive functions and schizophrenic symptoms, especially negative symptoms (Perlstein et al., 2001; Zhou et al., 2005; Ohtani et al., 2014). Furthermore, functional connectivity between the PFC and premotor and sensorimotor cortices decreased during the performance of the FEP task in schizophrenia (Chan et al., 2015b). In addition, the performance of the FEP task was correlated with PFC executive functions such as the WCST (present study) and performance in other tasks such as the Hayling sentence completion test in healthy adults (Varkovetski et al., 2020). These findings also support the present results that the performance of the FEP task reflects PFC executive functions, which are disturbed in schizophrenia.

The STA scores were correlated with performance errors in the FEP task, which is consistent with previous studies that reported an association between NSS or reduction of precision hand motor control and positive schizotypal personality (Lenzenweger and Maher, 2002; Barkus et al., 2006). However, the STA scores were not correlated with PFC hemodynamic response changes between the first and second trials of the FEP task, or with perseveration errors in the WCST. STA scores are known to reflect positive schizophrenic symptoms (Asai et al., 2008), while NSSs, including performance deficits in the FEP task, are associated with negative schizophrenic symptoms and cognitive impairments (Chan et al., 2015a; Herold et al., 2019). The differences between the FEP task and STA scores might explain the correlation differences in the PFC hemodynamic responses. Consistently, a previous fMRI study reported that higher positive schizotypal traits were associated with brain regions other than the PFC, such as the hippocampus (Modinos et al., 2018). These findings suggest that STA scores may be more strongly associated with functions in brain areas other than the PFC, while FEP task performance may be more strongly associated with the PFC functions.

## Conclusion

In conclusion, the present study indicated a significant correlation between learning (error reduction) in the FEP task and PFC hemodynamic responses in the early trials. Previous studies also reported that PFC activity increased initially during learning of non-motor cognitive tasks (Konishi et al., 2008; Jimura et al., 2014). It is noted that performance of the new hand motor sequence required learning (as shown by error reduction) in the FEP task, while the PT task required no learning. This suggests that the FEP task imposes higher cognitive load on the PFC than the PT task, consistent with higher hemodynamic activity in the first trial of the FEP task. Furthermore, the PFC has been implicated in learning of not only motor behaviors (Ishikuro et al., 2014; Ota et al., 2020; present study) but also various cognitive skills (Konishi et al., 2008; Cole et al., 2013; Jimura et al., 2014). The PFC may be a universal flexible hub to connect modules required for novel tasks (Cole et al., 2013). These findings suggest that the PFC might be involved in learning of the FEP task through its effects on the motor cortex. Further studies are required to investigate hemodynamic activity in the motor cortex and functional connectivity between the PFC and motor cortex during learning of the FEP task with high density NIRS recording (Ota et al., 2020). On the other hand, performance disturbance in the FEP task has been reported not only in schizophrenia but also in mild cognitive impairment, Alzheimer’s disease, and Parkinson’s disease (Fama and Sullivan, 2002; Chan and Gottesman, 2008; Weiner et al., 2011). Our findings suggest that the FEP task in conjunction with fNIRS may be a useful diagnostic method not only for PFC lesions but also for various disorders with PFC dysfunction, including schizophrenia, mild cognitive impairment, Alzheimer’s disease, and Parkinson’s disease.

## Data Availability Statement

The original contributions presented in the study are included in the article/Supplementary Material, further inquiries can be directed to the corresponding author.

## Ethics Statement

The studies involving human participants were reviewed and approved by the ethical committee at the University of Toyama. The patients/participants provided their written informed consent to participate in this study.

## Author Contributions

SK and HNj designed the experiments. SK and YI performed the experiments. SK, YI, and HNj analyzed the data and wrote the manuscript. SK, YI, HNm, JM, TS, TO, and HNj revised the manuscript. All authors discussed the results and approved the final manuscript.

## Conflict of Interest

The authors declare that the research was conducted in the absence of any commercial or financial relationships that could be construed as a potential conflict of interest.

## Footnotes

1 http://strokedatabank.ncvc.go.jp/archive/

2 http://bisp.kaist.ac.kr/NIRS-SPM

3 http://www.MRIcro.com
