# Supplementary material for: Examination of the Prefrontal Cortex Hemodynamic Responses to the Fist-Edge-Palm Task in Naïve Subjects Using Functional Near-Infrared Spectroscopy
Source: Front Hum Neurosci. 2021 Feb 5;15:617626. doi: 10.3389/fnhum.2021.617626 (PMC7901956; doi:10.3389/fnhum.2021.617626)
Supplement: Supplementary file 1 [file Presentation_1.pdf]

# Examination of the prefrontal cortex hemodynamic responses to the fist-edge-palm task in naïve subjects using functional near-infrared spectroscopy

## Supplementary information

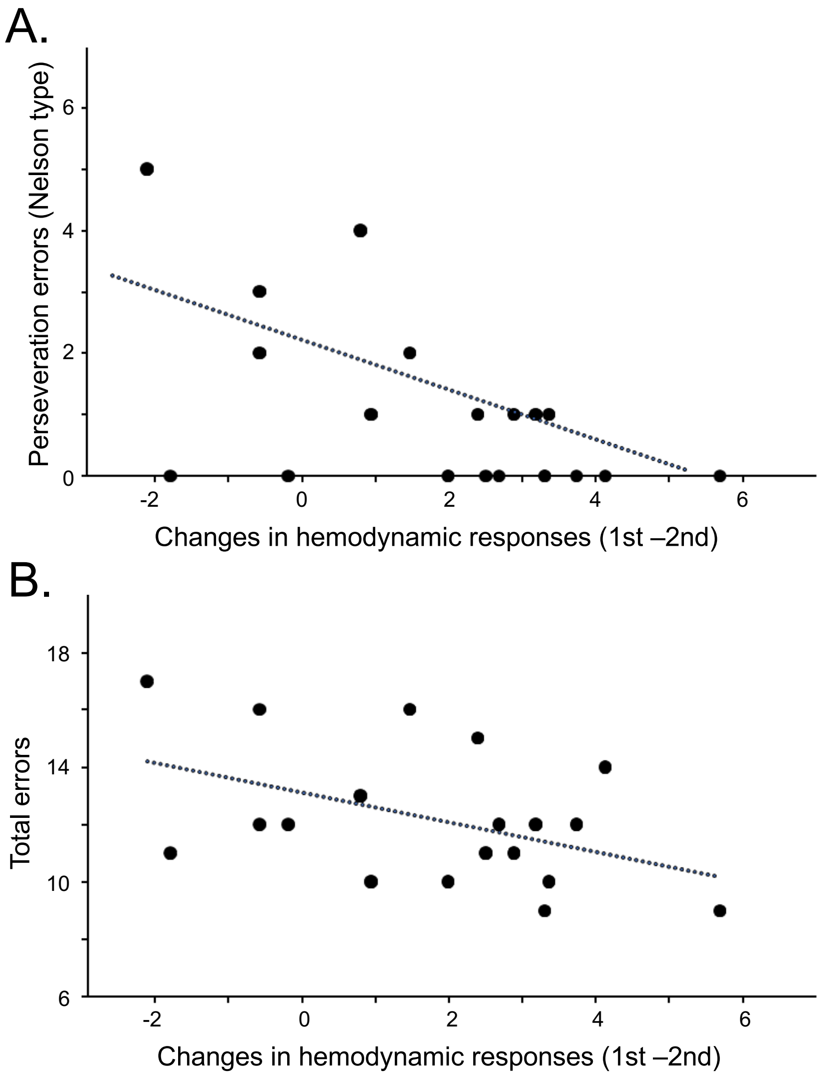


**Supplementary Figure 1. Relationships between the mean prefrontal cortex (PFC) hemodynamic responses in the Fist-Edge-Palm (FEP) task and Wisconsin card sorting test (WCST) data.**

A: Relationship between changes in the PFC hemodynamic responses (effect sizes) in the first and second trials and the number of perseveration errors of the Nelson type. Nelson type errors in the WCST were significantly and negatively correlated with the hemodynamic response changes ($r = -0.569$; $F[1, 18] = 8.155$, $p = 0.011$). Spearman's rank correlation test also revealed similar negative correlation ($rs = -0.517$, $p = 0.023$). B: Relationship between the changes in the mean PFC hemodynamic responses (effect sizes) between the first and second trials of the FEP task and total errors in the WCST. Total errors in the WCST tended to be negatively correlated to the hemodynamic response changes ($r = -0.454$; $F[1, 18] = 4.424$, $p = 0.051$). Spearman's rank correlation test also revealed similar negative correlation ($rs = -0.418$, $p = 0.075$).

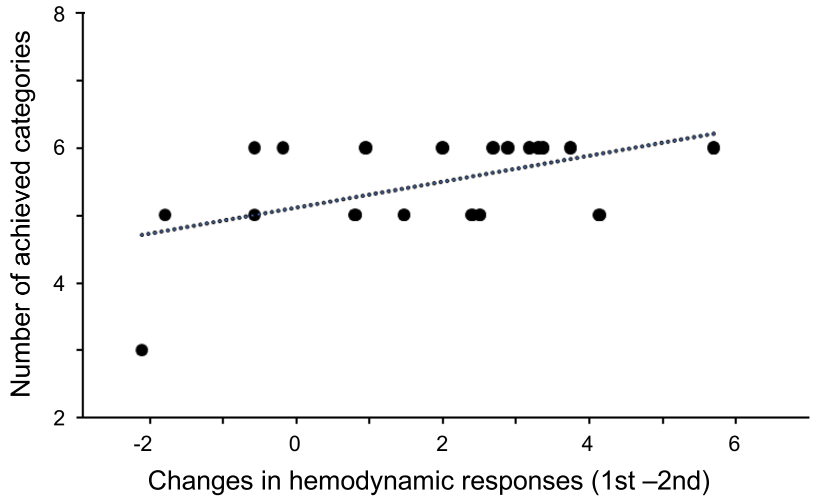


**Supplementary Figure 2. Relationships between the changes in the mean prefrontal cortex (PFC) hemodynamic responses (effect sizes) between the first and second trials and the number of achieved categories in the Wisconsin card sorting test (WCST).**

The number of achieved categories in the WCST was significantly and positively correlated to the hemodynamic response changes ($r = 0.522$; $F[1, 18] = 6.370$, $p = 0.022$). Spearman's rank correlation test also revealed similar tendency ($rs = 0.435$, $p = 0.063$).
